# Activation of quiescent polypoidal choroidal vasculopathy after membrane peeling vitrectomy for epiretinal membrane: a case report

**DOI:** 10.1186/s12886-021-02080-5

**Published:** 2021-09-06

**Authors:** Yuelin Wang, Zhe Chen, Weihong Yu, Youxin Chen

**Affiliations:** 1 Department of Ophthalmology, Peking Union Medical College Hospital, Chinese Academy of Medical Sciences, 100730 Beijing, China; 2 Key Lab of Ocular Fundus Disease, Chinese Academy of Medical Sciences, 100730 Beijing, China

**Keywords:** Polypoidal choroidal vasculopathy, Epiretinal membrane, Vitrectomy, Case report

## Abstract

**Background:**

Regular membrane peeling vitrectomy for epiretinal membrane (ERM) patients seldom causes large pigment epithelial detachment (PED). We presented an unusual case of the activation of quiescent polypoidal choroidal vasculopathy (PCV) after membrane peeling vitrectomy for ERM, with an uneven therapeutic process.

**Case presentation:**

A 75-year-old female patient complained of metamorphopsia in her left eye for 2 years. Her best-corrected visual acuity was 20/160 with a moderate nuclear cataract. An irregular ERM and slight PED were shown in optical coherence tomography (OCT). No obvious orange-red lesion was detected. The patient underwent vitrectomy + ERM peeling + cataract surgery. After the operation, large PED emerged, and indocyanine green angiography (ICGA) confirmed PCV. Four monthly injections of intravitreal ranibizumab were administered, but PED persisted. After focal laser therapy targeted to the polyps combined with ranibizumab treatment, PED was absorbed.

**Conclusions:**

Careful evaluation for PCV before membrane peeling vitrectomy for ERM is important, as indolent PCV may be activated postoperatively. Anti-VEGF therapy accompanied by laser photocoagulation may be more effective for PCV polyps located away from the fovea.

**Supplementary Information:**

The online version contains supplementary material available at 10.1186/s12886-021-02080-5.

## Background

Epiretinal membrane (ERM) refers to the fibrocellular proliferation on the surface of the neurosensory retina [1], which mostly occurs in people aged over 50, with a prevalence rate ranged from 2.2 to 9 % among the different populations [2]. The majority of ERMs will remain stable and do not require therapy. The decision to intervene usually depends on the severity of the patient’s symptoms, such as how much they are bothered by their visual dysfunction. Early surgical intervention enables long-term visual recovery than delayed [3]. At present, vitrectomy combined with inner limiting membrane (ILM) peeling is considered to be an effective treatment option for ERM. By peeling the ERM and ILM, the traction of the macula can be relieved, the anatomic structure of the macula can be improved, which results in better visual function with a lower recurrence rate of ERM [4].

Regular ILM peeling vitrectomy seldom causes large pigment epithelial detachment (PED). We presented an unusual case of the activation of quiescent polypoidal choroid vasculopathy (PCV) after membrane peeling vitrectomy with ineffective monotherapy with anti-VEGF drugs. This report was organized in adherence to CARE guidelines (see Supplementary File 1) [5].

## Case presentation

**Fig. 1:**
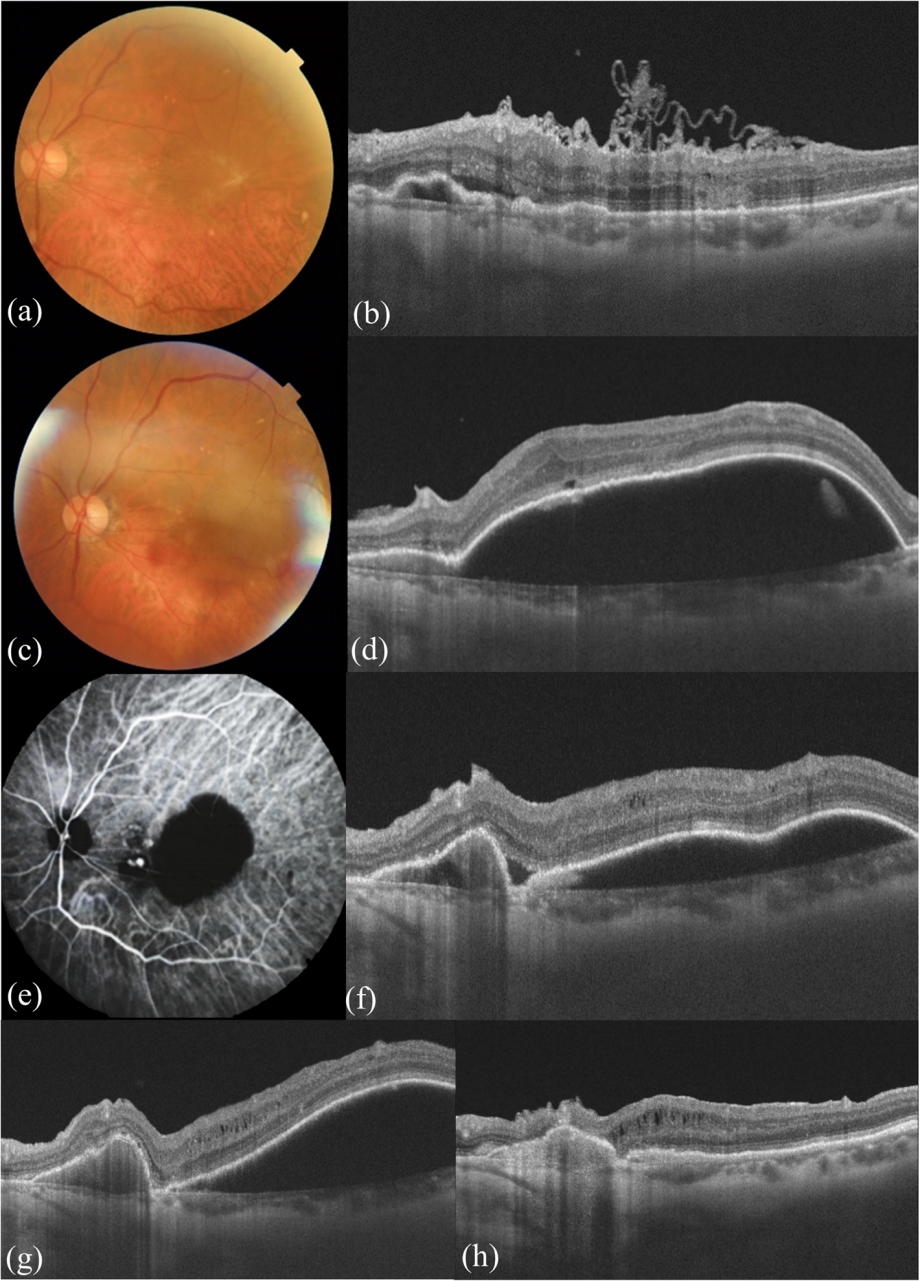
**a** Fundus photograph showed the greyish-white ERM. **b** OCT (10 o’clock position) showed an irregularly folded epiretinal membrane (the “spaghetti sign”) with edema. The nasal side of the macula showed a slight PED and a small amount of subretinal fluid. **c**,**d** One month after vitrectomy with ERM peeling, a significant increase in the height of PED was shown in the fundus and OCT, with orange polyps near the sub-temporal vessels. **e** ICGA showed focal hyperfluorescent lesions at the nasal border of the PED lesion. **f** After one injection of intravitreal ranibizumab, the height of the PED was decreased. **g** After four monthly injections of intravitreal ranibizumab, PED persisted. **h** After focal laser therapy targeted to the polyps combined with ranibizumab, PED was absorbed

A 75-year-old female patient presented with metamorphopsia and decreased vision in her left eye for 2 years. She denied any history of diabetes or hypertension. Left eye examination: best-corrected visual acuity was 20/160, and slit-lamp examination revealed a moderate nuclear cataract. An irregular dense ERM (the “spaghetti sign”) with macular edema and slight PED were detected on optical coherence tomography (OCT). No obvious orange-red lesions or hemorrhages were seen. (see Fig. 1a and b).

The patient was diagnosed with ERM, wet age-related macular degeneration (wAMD), macular pucker, and age-related cataract in her left eye. The patient underwent phacoemulsification + pars plana vitrectomy + ILM peeling + intraocular lens implantation in the left eye, and the operation procedure was uneventful.

One month later, the visual acuity in her left eye was 20/80, and OCT showed significant PED with subretinal fluid (see Fig. 1c and d). Indocyanine green angiography (ICGA) showed a few polyps and adjacent large PED (see Fig. 1e) at the macula. PCV of the left eye was diagnosed afterward. After one injection of intravitreal ranibizumab, the height of the PED was decreased (see Fig. 1f), but after another three consecutive monthly intravitreal anti-VEGF drug (ranibizumab) injections, PED was not resolved (see Fig. 1 g). After laser photocoagulation combined with ranibizumab, the PED was absorbed (see Fig. 1 h), and visual acuity improved to 20/63. The patient was satisfied with her present treatment.

## Discussion and conclusions

PCV is a disease characterized by enlarged choroidal nodular lesions and abnormal branching of the choroidal vascular network, which is more common in Asians. Orange-red polyps are often detected, sometimes with subretinal hemorrhage, exudation, and PED [6]. However, some polyps may be ignored when PCV is indolent or combined with cataracts. According to the EVEREST criteria, focal hyperfluorescent lesions appearing before 6 min on ICGA is a necessary criterion for PCV diagnosis [7]. Therefore, careful inspection of the fundus with the ICGA exam was required.

The “spaghetti sign” refers to ILM dehiscence, which presents as hyporeflective linear spaces of varying lengths and depths interspersed with hyper-reflective bands, and protrusions of retinal nerve fiber layer (RNFL) tissue projecting into the posterior vitreous cavity on OCT [8, 9]. The “spaghetti sign” sometimes appears because of posterior vitreous detachment, which creates a longitudinal pulling force on the ILM and RNFL but with residual tight adhesion. Vitrectomy and ILM peeling may pull the retina and cause tractional complications, such as PED or even retinoschisis. Therefore, the “spaghetti sign” may serve as a warning sign for membrane peeling vitrectomy.

But for this patient, PCV was quiescent preoperatively, vitrectomy had activated PCV with unknown mechanisms. We hypothesized that (1) intraocular pressure fluctuating during the operation could damage the choroidal vascular walls of polyps, thus disturbing the stability of the indolent PCV lesion; (2) after the vitreous was removed and the ILM was peeled, the restricting force on the retina was lost, which enabled the PCV lesions to inflate and activate; (3) during the membrane peeling vitrectomy operation process, iatrogenic strength may create an upward pulling force on the whole retina, especially with ERM (the “spaghetti sign”), which causes the junctional structure of the retinal pigment epithelium (RPE) and photoreceptor cells to loosen, and may lead to subretinal fluid effusion and large PED formation; (4) Vitrectomy can lead to a series of inflammatory responses, with the release of VEGF, PIGF, interleukin, and other inflammatory factors. Vascular permeability was changed, subretinal fluid was aggregated, and PED occurred. For our case, the presence and inactive nature of the PCV was not confirmed pre-operatively with ICGA, it is not known whether the subsequent surgery had any effect on the PCV. The development of actively leaking polyps may have simply been a coincidence rather than any direct effect of the surgery. Hence, ICGA should be used to evaluate PCV activity before membrane peeling vitrectomy.

For the treatment of PCV, anti-VEGF therapy is an important treatment choice. For this patient, using ranibizumab, a small-molecule antibody fragment, partially reduced her PED after the first injection, but after the second, third, and fourth monthly injections, PED persisted. At present, some researchers have reported the different effects of the fusion protein and small-molecule antibody fragment anti-VEGF drugs in the treatment of PED. Hata et al. conducted a retrospective study on 216 patients with AMD and found a greater reduction in PED in the aflibercept group than in the ranibizumab group [10]. It is worth of note that anti-VEGF agents approved in clinical practice, such as ranibizumab and aflibercept, are considerably different in terms of molecular interactions when they bind with VEGF [11]; therefore, characterization of such features can improve the design of novel biological drugs potentially useful in clinical practice. Other researchers suggest that [12] aflibercept reduced the thickness of the choriocapillaris in monkey eyes more significantly than ranibizumab, possibly because of an interaction between the Fc fragment and other molecules. Additional binding of aflibercept to placental growth factor (PlGF) enabling higher binding affinity to VEGF than other VEGF inhibitors, and the development of autoantibodies before anti-VEGF therapy could also serve as potential explanations [13]. Therefore, fusion protein drugs can be a choice of alternatives for the treatment of refractory PED.

For this patient, considering that ranibizumab did not have an obvious therapeutic effect, laser photocoagulation was combined. The therapeutic target of laser photocoagulation is the position of high fluorescence on ICGA. After laser photocoagulation combined with ranibizumab, PED decreased significantly. For a long time, focal laser therapy has been used to ablate extrafoveal and extramacular polyps identified on ICGA. Focal laser therapy in combination with anti-VEGF therapy has been described to be effective in eyes with extrafoveal PCV. Lee et al. [14] reported stable vision or visual improvement in 75 % of PCV patients, while Yuzawa et al. [15] demonstrated a decrease in visual acuity in 54 % of PCV patients. The use of lasers may cause choroidal scars and subretinal or sub-RPE hemorrhage, which may influence vision. However, the combination of anti-VEGF and laser therapy was reported to improve vision [16]. Compared with photodynamic therapy (PDT), laser photocoagulation is cheaper, more reachable, and has a similar therapeutic effect for PCV [17]. Therefore, laser therapy can also be effective for PCV treatment, especially with the combination of anti-VEGF drugs.

In conclusion, careful evaluation for PCV by ICGA before membrane peeling vitrectomy for ERM is important, as indolent PCV may be activated postoperatively. Fusion protein anti-VEGF drugs may be preferred for PED therapy, and anti-VEGF accompanied by laser photocoagulation may be effective for PCV polyps located away from the fovea.

## Supplementary Information

**Additional file 1.**

## Data Availability

The datasets used and/or analyzed during the current study are available from the corresponding author on reasonable request.

## Abbreviations

ERM: Epiretinal membrane
ICGA: Indocyanine green angiography
ILM: Internal limiting membrane
OCT: Optical coherence tomography
PED: Pigment epithelial detachment
PCV: Polypoidal choroidal vasculopathy
PDT: Photodynamic therapy
RNFL: Retinal nerve fiber layer
wAMD: Wet age-related macular degeneration
